# Cloning, expression analysis, and RNA interference study of a HORMA domain containing autophagy-related gene 13 (ATG13) from the coleopteran beetle, *Tenebrio molitor*

**DOI:** 10.3389/fphys.2015.00180

**Published:** 2015-06-17

**Authors:** Jung Hee Lee, Yong Hun Jo, Bharat Bhusan Patnaik, Ki Beom Park, Hamisi Tindwa, Gi Won Seo, Raman Chandrasekar, Yong Seok Lee, Yeon Soo Han

**Affiliations:** ^1^Division of Plant Biotechnology, Institute of Environmentally-Friendly Agriculture, College of Agriculture and Life Sciences, Chonnam National UniversityGwangju, South Korea; ^2^Trident School of Biotech Sciences, Trident Academy of Creative TechnologyBhubaneswar, India; ^3^Department of Biochemistry and Molecular Biophysics, Kansas State UniversityManhattan, KS, USA; ^4^Department of Life Science and Biotechnology, College of Natural Sciences, Soonchunhyang UniversityAsan, South Korea

**Keywords:** *Tenebrio molitor*, autophagy, ATG13, HORMA domain, expression analysis, RNA interference

## Abstract

Autophagy is a process that is necessary during starvation, as it replenishes metabolic precursors by eliminating damaged organelles. Autophagy is mediated by more than 35 autophagy-related (Atg) proteins that participate in the nucleation, elongation, and curving of the autophagosome membrane. In a pursuit to address the role of autophagy during development and immune resistance of the mealworm beetle, *Tenebrio molitor*, we screened ATG gene sequences from the whole-larva transcriptome database. We identified a homolog of ATG13 gene in *T. molitor* (designated as TmATG13) that comprises a cDNA of 1176 bp open reading frame (ORF) encoding a protein of 391 amino acids. Analyses of the structure-specific features of TmAtg13 showed an intrinsically disordered middle and C-terminal region that was rich in regulatory phosphorylation sites. The N-terminal Atg13 domain had a HORMA (Hop1, Rev7, and Mad2) fold containing amino acid residues conserved across the Atg13 insect orthologs. A quantitative reverse-transcription-polymerase chain reaction analysis revealed that TmATG13 was expressed ubiquitously during all developmental stages of the insect. TmATG13 mRNA expression was high in the fat body and gut of the larval and adult stages of the insect. The TmATG13 transcripts were expressed at a high level until 6 days of ovarian development, followed by a significant decline. Silencing of ATG13 transcripts in *T. molitor* larvae showed a reduced survivability of 39 and 38% in response to *Escherichia coli* and *Staphylococcus aureus* infection. Furthermore, the role of TmAtg13 in initiating autophagy as a part of the host cell autophagic complex of the host cells against the intracellular pathogen *Listeria monocytogenes* is currently under study and will be critical to unfold the structure-function relationships.

## Introduction

Eukaryotic cells have developed efficient mechanisms to cope with environmental stressors, including the clearance of harmful pathogens from the cytosol. Autophagy is a conserved mechanism, whereby the cytoplasmic contents are degraded in the lysosomes. The sequestration of the cytoplasm forms the developing autophagosome that finally fuses with a lysosome in the cytoplasm, and the cytoplasm-derived material is degraded by lysosomal hydrolases (Nakatogawa et al., 2009; Rubinsztein et al., 2012). The breakdown products are recycled to allow further metabolism needed for survival. Autophagy was first identified as a cellular response to nutrient deprivation. Since then, it has been implicated in a multitude of developmental processes including tissue remodeling during development (Tsukamoto et al., 2008), innate immune responses (Levine and Deretic, 2007), and disease-related functions as in aging, cancer, and neurodegenerative diseases (Mizushima et al., 2008).

The autophagy pathway, particularly the formation of autophagosome, is mediated by autophagy-related (ATG) genes. These genes code for approximately 35 or so specialized Atg proteins, first identified in yeast through genetic screening and have now been implicated in the autophagy mechanisms of higher eukaryotes (He and Klionsky, 2009). The regulatory function of autophagy in insects attributed to PI3K-AKT/PKB-TSC1/TSC2-Rheb-TOR signaling pathway (Rusten et al., 2004). The pathway is activated by 20-hydroxyecdysone (20E) and is mediated by transcription factors, such as β FTZ-F1, BR-C, E74A, and E93. The mTOR complex inhibits the activity of Atg1/Atg13 protein kinase complex under normal conditions; thus, acting as a negative regulator of autophagy (Ravikumar et al., 2004; Malagoli et al., 2010; Yeh et al., 2011). Autophagy in *Saccharomyces cerevisiae* is controlled by the cAMP-dependent protein kinase (PKA) pathway, which directly phosphorylates Atg13, acting as a conserved regulator of Atg1 kinase activity (Funakoshi et al., 1997; Hosokawa et al., 2009; Stephan et al., 2009). mTOR activity declines under nutrient-deprived conditions leading to the nucleation of the autophagosome membrane via the formation of the Atg1/Atg13 and autophagy-related gene 6- Vps34 (also called phosphatidylinositol 3-kinase)—immune response deficient1 (Atg6-Vps34-Ird1) complexes. Furthermore, the role of mTOR in autophagy has been established through elegant genetic studies (Sancak et al., 2010), although the exact mechanism remains unknown. During the process of elongation of phagophore membrane in insects, the Atg8-phosphatidylethanolamine and the Atg12-Atg5 conjugated protein complex, becomes integral part of the membrane. The Lqf/Epsin protein is needed towards curving of the autophagosome membrane. These highly curved vesicles are recognized by the Atg1 C-terminal early autophagy targeting/tethering (EAT) domain (Chang and Neufeld, 2009; Ragusa et al., 2012).

The protein kinase Atg1 and the phosphoprotein Atg13 functions as a critical component in nutrient-dependent autophagic signaling. Although the complex has been sufficiently studied for its presumptive roles of autophagy in higher eukaryotes and metazoans (Chan et al., 2009; Jung et al., 2009; Alers et al., 2011), its regulation of the autophagic machinery in arthropods including insects is less known. Atg13 in *Drosophila* stimulates both autophagic activity of Atg1 and its inhibition of cell growth and TOR signaling that maintains a regulatory link between TOR and the Atg1/Atg13 complex, which is very similar to yeast and metazoans (Scott et al., 2007; Chang and Neufeld, 2009). Until now, Atg1 and Atg13 phosphorylation mechanism have not been clearly defined. One study reported that mTOR regulates phosphorylation of Atg13, ULK1 and ULK2 (which are mammalian homologs of Atg1). Thus, Atg13 functions in mammals to stabilize ULK interactions and facilitate the FIP200 phosphorylation by ULK (Jung et al., 2009). An understanding of the structure of insect Atg13 will be useful to understand the molecular mechanisms of autophagy initiation at phagophore assembly site (PAS). The Atg13 HORMA (Hop1p, Rev1p, and Mad2) domain, which is similar to the spindle checkpoint protein Mad2, has been mapped at the structural level which provided insight into its mediation of autophagy (Jao et al., 2013b).

The yellow mealworm, *Tenebrio molitor* is a useful model for studying insect biochemistry, immunology, and physiology due to its large size, ease of handling, and compatible genetics (Jiang et al., 2011). We have screened and characterized immune genes from this insect using expressed sequence tag (EST) and RNA sequence (RNAseq) analysis (Jeong et al., 2013; Patnaik et al., 2013, 2014; Tindwa et al., 2013; Noh et al., 2014). We have also screened and identified the autophagy genes from *T. molitor* using the RNA-seq approach. We understand that autophagy research in insects is in its infancy compared to that of the well-characterized models in yeast, *Drosophila*, and mammals. The present research aims at establishing a more general model for insects that may or may not be in agreement with the Dipteran findings. Herein, we hypothesized a necessary role for the Atg13 ortholog in *T. molitor* larvae during initiation of autophagy and a function during ovary maturation and development. The TmATG13 gene was characterized at the sequence and structure level using molecular cloning and bioinformatics approaches. The folded structure of the deduced HORMA domain protein was mapped using homology modeling and structural prediction software's. We also assessed the developmental and tissue-specific mRNA expression of TmATG13 and its relevance during ovarian maturation and development. Furthermore, the functional significance of the TmATG13 transcripts using gene silencing study elaborated on its requirement for survivability against bacterial infections.

## Materials and methods

### Insect rearing and maintanence

*T. molitor* larvae were maintained in an environmental chamber at 26 ± 1°C with 60 ± 5% relative humidity. The larvae were fed an autoclaved artificial diet composed of 220 g wheat bran (Milworm House, Busan, Korea), 10 g bean powder (Donggran Food, Daejeon, Korea), 5 g brewer's yeast (Beer Yeast Korea Inc., HNH Seoul, Korea), 0.15 g chloramphenicol (Duchefa Biochemie, Haarleem, The Netherlands), 1.1 g sorbic acid (Sigma Aldrich, St. Louis, MO, USA), and 1.1 ml propionic acid (Sigma Aldrich, Louis, USA) mixed in 430 ml of distilled water.

### Cloning of the cDNA encoding TmATG13

**Figure 1:**
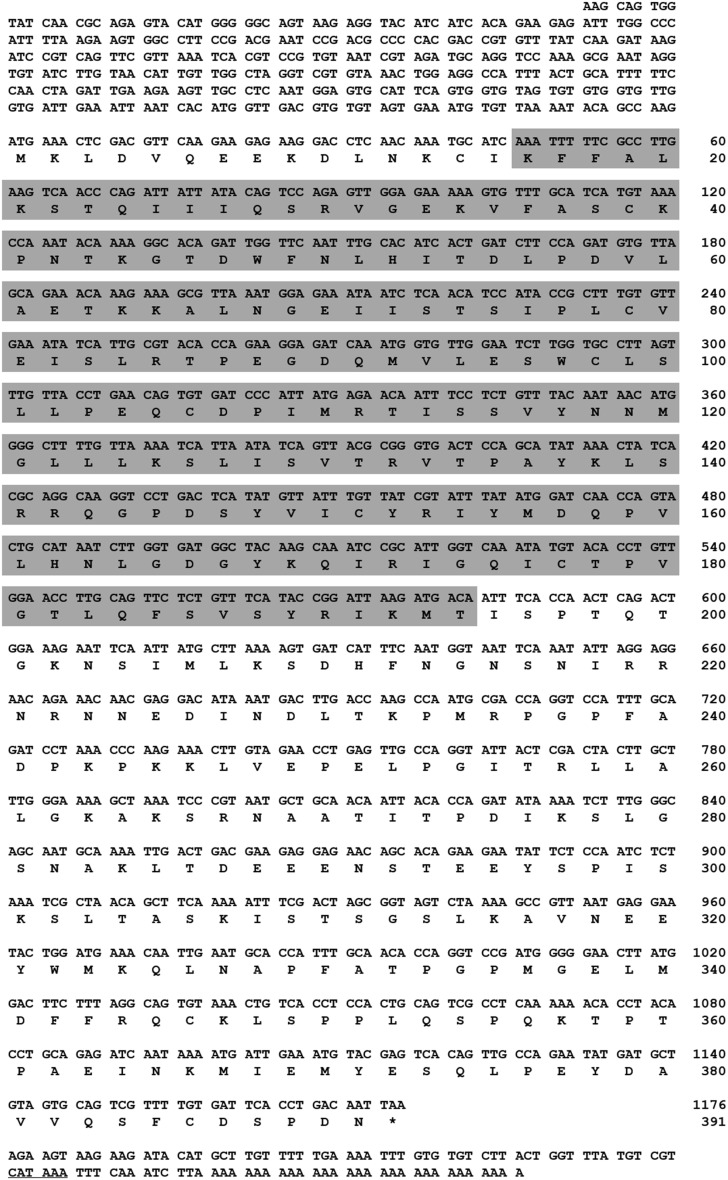
**Nucleotide and deduced amino acid sequence of TmAtg13 cDNA**. The full-length ORF sequence was derived from the *Tenebrio molitor* transcriptome database. The 5′/3′-UTR region was obtained by RACE-PCR methods. Asterisk denotes the stop codon. The polyadenylation domain in the 3′-UTR is underlined. Gray box indicates the N-terminal HORMA domain.

Reverse transcription- polymerase chain reaction (RT-PCR) and 5′/3′ rapid amplification of cDNA ends- PCR (RACE-PCR) analyses were used to clone the full-length open reading frame (ORF) of TmATG13, identified from RNAseq analysis of *T. molitor* larvae. All primers were synthesized using the PrimerQuest program (http://www.idtdna.com/Scitools) and are detailed in Figure 1. Briefly, the synthesized cDNAs from last-instar *T. molitor* larvae were amplified using RT-PCR and the pre-designed primers under the following conditions: denaturation at 94°C for 30 s, annealing at 48°C for 30 s, and extension at 72°C for 30 s for 30 cycles. The amplified gene products were purified using an AccuPower PCR purification kit (Bioneer, Daejeon, Korea), according to manufacturer's instructions. The purified PCR products were incubated at 72°C for 15 min to produce sticky or cohesive ends using Taq polymerase. Subsequent to ligation into the pCR2.1 TOPO TA cloning vector (Invitrogen Corporation, Carlsbad, CA, USA) and transformation into *Escherichia coli* cells, the cloned insert was sequenced using the 454 genome sequencer-GS FLX+ system (Roche Life Sciences, Branford, CT, USA).

The 5′/3′ extensions of the TmAtg13 ORF sequence were obtained using the SMARTer RACE cDNA amplification kit (Clontech Laboratories, Palo Alto, CA, USA) according to the manufacturer's instructions. The RACE conditions were as follows: pre-denaturation at 98°C for 5 min, denaturation at 98°Cfor 10 s, annealing at 55°C for 30 s, and extension at 72°C for 1 min. The nested PCR products were purified and subsequently cloned and sequenced as described above.

The TmAtg13 cDNA sequence has been registered in GenBank with the accession number KJ778621.

### Sequence alignment, domain analysis, and phylogenetic tree

The deduced amino acid sequence corresponding to the N-terminal conserved HORMA domain was analyzed in detail. The specific domain was predicted using the InterProScan (http://www.ebi.ac.uk/Tools/pfa/iprscan/) and BLAST programs at NCBI (http://blast.ncbi.nlm.nih.gov/). The TmAtg13 orthologous sequences for multiple alignment were derived from GenBank and analyzed using Clustal W2 (http://www.ebi.ac.uk/Tools/msa/clustalw2/) and GeneDoc ver. 2.7 (http://www.psc.edu/).

The structure representing the N-terminal HORMA domain was obtained by constructing a homology model using the SWISS MODEL workspace (http://swissmodel.expasy.org/workspace). The predicted model was based on a reference template from the Protein Data Bank (PDB ID: 4J2G at 2.29 Å resolution). The predicted model was visualized using the PyMOL ver. 1.5 software (http://www.pymol.org/). Additionally the 3D structure of Human mitotic spindle assembly checkpoint protein MAD2A was retrieved from the protein data bank (PDB ID: 1KLQ; DOI: 10.2210/pdb1klq/pdb). The structural identities were assigned to the multiple sequence data using the protein structure database DaliLite ver. 3.0 (Holm and Rosenstrom, 2010).

The evolutionary position of the TmAtg13 N- terminal HORMA domain was analyzed with respect to its orthologs using the maximum likelihood method based on the Jones -Thornton -Taylor (JTT) model (Felsenstein, 1981). A bootstrap consensus tree of 1000 replicates was used to evaluate branch strength for analysis using MEGA ver. 6.06 (Tamura et al., 2013).

### QRT-PCR

The Atg13 mRNA expression was analyzed in the larval and adult tissues of *T. molitor* using qRT-PCR. For the same, the last-instar larval tissues and 2-day old adult tissues, including the fat body, gut, hemocytes, integument, Malphigian tubules, ovary, and testes were dissected from healthy insects. For developmental expression analysis, healthy *T. molitor* samples from the last-instar larvae, pre-pupa, day 1–7 pupa, and adult day 1 and day 2 samples were used. Ovaries (*n* = 3) were dissected from adult females on alternate days and used for total RNA isolation and cDNA synthesis to evaluate TmAtg13 expression during ovarian maturation. The qRT-PCR analysis was performed using the Bioneer Exicycler 96 Real-Time Quantitative Thermal Block and SYBR Green-containing qPCR Master Mix. Total RNA was isolated from tissues and developmental samples using the SV isolation system (Promega Corp. Madison, WI, USA). cDNA was generated from 1 μg of total RNA with oligo (dT) primers and the AccuPower RT premix (Bioneer). The resulting cDNAs were applied to qRT-PCR analyses and amplified using primers (Table 1) under a standard temperature profile (pre-denaturation at 95°C for 20 s, followed by 40 cycles denaturation at 94°C for 5 s, annealing and extension at 60°C for 20 s). Each analysis was measured independently at least three times. The 2^−ΔΔCt^ method (Livak and Schmittgen, 2001) was employed to analyze the TmAtg13 expression levels. The gene expression levels were normalized to *T. molitor* ribosomal protein L27a (TmL27a), which served as an internal control. Data are presented as relative mRNA expression levels (mean ± standard deviation, *n* = 3).

**Table 1:** **Primers used in the present study**.

**Name**	**Primer sequences**
TmAtg13 5′-RACE GSP1 TmAtg13 5′-RACE GSP2	5′-GGTTGACTTCAAGGCGAAAA-3′ 5′-AGGCACCAAGATTCCAACAC-3′
TmAtg13 3′-RACE GSP1 TmAtg13 3′-RACE GSP2	5′-GAATGCACCATTTGCAACAC-3′ 5′-AAAATCTTTGGGCAGCAATG-3′
TmAtg13 qPCRFw TmAtg13 qPCRRv	5′-ACCATTTGCAACACCAGGTC-3′ 5′-TTTTTGAGGCGACTGCAGTG-3′
TmL27a qPCRFw TmL27a qPCRRv	5′-TCATCCTGAAGGCAAAGCTCCAGT-3′ 5′-AGGTTGGTTAGGCAGGCACCTTTA-3′
dsTmAtg13 Fw dsTmAtg13 Rv dsEGFP Fw dsEGFP Rv	5′-TAATACGACTCACTATAGGGTGTGTTGGAATCTTGGTGCCT-3′ 5′-TAATACGACTCACTATAGGGTGTTGCAGCATTACGGGATTT-3′ 5′-TAATACGACTCACTATAGGGTACGTAAACGGCCACAAGTTC-3′ 5′-TAATACGACTCACTATAGGGTTGCTCAGGTAGTGGTTGTCG-3′

Polymerase recognition signals have been underlined.}

### RNA interference (RNAi) of TmATG13

The dsRNA fragments of TmATG13 gene were synthesized for silencing the corresponding mRNA transcripts. For the same, TmATG13 DNA fragment was amplified from cDNA by PCR using gene-specific primers tailed with a T7 promoter sequence (Table 1). The PCR reaction was carried out under the following conditions: initial denaturation at 94°C for 3 min, followed by 28 cycles of denaturation at 94°C for 30 s, annealing at 56°C for 30 s, extension at 72°C for 1 min, and a final extension at 72°C for 10 min. The PCR product was purified using the AccuPrep PCR Purification kit (Bioneer, Daejeon, South Korea) and was used to synthesize the double-stranded RNA (dsRNA) using Ampliscribe™ T7-Flash™ Transcription kit (Epicentre Biotechnologies, Madison, WI, USA). The dsRNA product was purified and precipitated with 5 M ammonium acetate. Integrity of the purified dsRNA was confirmed by running 1% agarose gel electrophoresis and quantified using a NanoDrop spectrophotometer (Thermo Scientific, Wilmington, DE, USA). dsRNA for the enhanced green fluorescent protein (dsEGFP) was synthesized in the same way, and all dsRNAs were stored at −20°C until injection into *T. molitor* larvae.

The dsTmATG13 or dsEGFP (1 μg/larva) were dissolved in water and injected into the hemocoel of the larvae (*n* = 20) using disposable needles mounted onto a micro-applicator (Picospiritzer III Micro Dispensing System, Parker Hannifin, Hollis, NH, USA). Animals treated with the dsRNAs were maintained on an artificial diet as described above.

### Bacterial injections and bioassays

*E. coli* K12 and *Staphylococcus aureus* RN4220 were grown in Luria Bertani broth (LB) [10 g tryptone, 5 g yeast extract, 10 g sodium chloride (pH, 7.0) per liter]. Aliquots of 10^6^ cfu/larva of *S. aureus* or *E. coli* were injected into *T. molitor* larvae (*n* = 20) to investigate the TmAtg13 induction pattern in response to the microbial injections. Total RNAs and cDNAs were prepared from samples of each treatment taken at 3, 6, 9, and 12 h post-injection. Relative TmAtg13 expression levels at different time points were determined by the qPCR, as described above.

To investigate the role of TmATG13 in larval survival against *S. aureus and E. coli* infections, the larvae were injected with either dsEGFP or dsTmATG13. The larvae were challenged with 10^6^ cfu/larva of *S. aureus* or *E. coli* 4 days after the dsRNA injection. Treated larvae were incubated at 26°C, and the number of dead larvae was recorded on a daily basis for 7 days post-injection. Survival rates of survival were compared between the dsTmATG13 and dsEGFP-treated groups. Statistical analysis was conducted by Wilcoxon-Mann-Whitney test and the cumulative survival rates were considered significant (*P* < 0.05).

## Results

### Identification of the cDNA encoding TmATG13 and domain architecture

We identified the full-length TmATG13 ORF from a *T. molitor* larval transcriptome database. The full-length TmATG13 cDNA including the 5′/3′- untranslated regions (UTRs) was identified using RACE-PCR approach. The TmAtg13 nucleotide and deduced amino acid sequences are shown in Figure 1. The *T. molitor* ATG13 gene and protein of *T. molitor* were designated as TmATG13, and TmAtg13, respectively. The sequence analysis showed that the TmATG13 cDNA had a 1176 bp ORF encoding a protein of 391 amino acid residues. The 5′-untranslated region contains 369 bp, and the 3′-UTR contained 106 bp ending with a 29 bp polyadenylation (A)^+^ (polyA^+^) tail. The ORF predicted a protein with a molecular mass of 43.7 kDa and a pI of 8.32.

**Figure 2:**
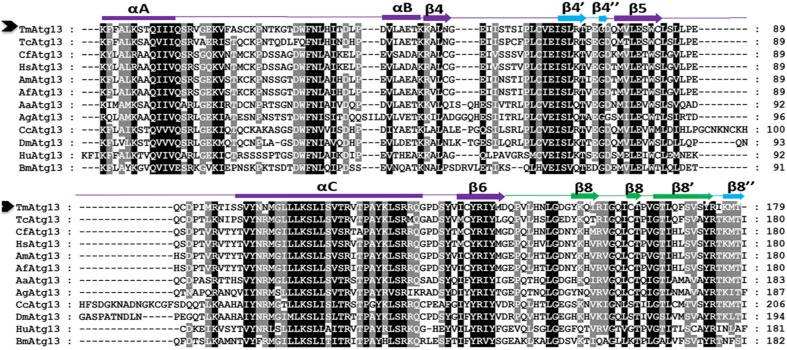
**Multiple alignment of the deduced amino acid sequence of the TmAtg13 protein HORMA domain with its orthologs**. The secondary structural elements predicted by a homology model (reference template PDB id: 4j2gA) and DaliLite ver. 3.0 are represented on the top of the sequence. Regions structurally similar to MAD2 are colored in purple except the safety belt region, which is highlighted in green. The Atg13 HORMA domain (β4′, β4″, andβ8″), which is unique to TmAtg13, is colored in blue. The amino acid residues that are identical or similar in all sequences are shaded black, gray sequences indicate identical or similar amino acids in most sequences. Dashes indicate gaps to optimize the alignment.

A search of the protein database using Basic Local Alignment Search Tool (BLAST) revealed high sequence similarities between TmAtg13 and other Atg13 orthologs from other insects. Analyses of the TmAtg13 deduced amino acid sequence revealed a highly conserved N-terminal Atg13 domain and an unstructured and variable C-terminal region. The Atg13 domain extended from amino acids 16–194 (Figure 1). Detailed analyses of TmAtg13 Atg13 domain using predictive sequence and structure analyses showed that the N-terminal domain belonged to the HORMA family. Multiple sequence alignment with conserved residues and the secondary structural elements corresponding to the TmAtg13 HORMA domain are shown in Figure 2.

**Figure 3:**
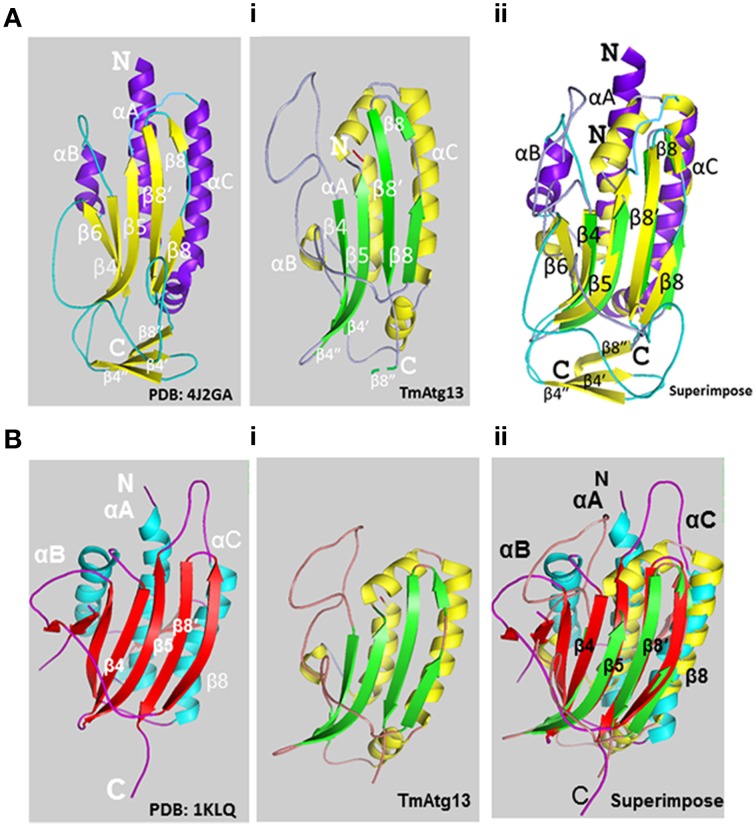
**Molecular structure of the TmAtg13 HORMA domain. (Ai)** The predicted structural model of TmAtg13 N-terminal HORMA domain is shown in parallel to the reference 3D-model of *Lachancea thermotolerans* Atg13 (PDB id: 4J2GA). **(Aii)** Superposed 3D-image of the N-terminal HORMA domain of TmAtg13 over the reference Atg13 model of *Lachancea thermotolerans* (PDB: 4J2GA). The alpha-helices and beta-sheets for the reference model and TmAtg13 HORMA domain are represented in purple and yellow, and yellow and green, respectively. **(Bi)** The predicted structural model of TmAtg13 N-terminal HORMA domain is shown in parallel to the 3D-model of Human mitotic assembly check-point protein MAD2 (PDB id: 1KLQ). **(Bii)** Superposed image of TmAtg13 HORMA domain with the Human MAD2 protein. The alpha-helices and beta-sheets are represented in cyan and red, and red and yellow, respectively for the reference Human MAD2 protein and TmAtg13 N-terminal HORMA domain.

The HORMA domain homology model of *T. molitor* Atg13 was built based on the reference Atg13 HORMA domain of *Lachancea thermotolerans* (LtAtg13; PDB id: 4J2G) (Jao et al., 2013a). Figure 3Ai shows the three-dimensional (3D) molecular structure of the HORMA domain of LtAtg13 and TmAtg13, respectively. The superimposed 3D structure of TmAtg13 (Figure 3Aii) showed structural homology in the core region, with large variations in the outside loop region. The prediction analysis of the HORMA domain's secondary structure suggested a globular protein that could potentially form a complex β-sheet(s) with associated α-helices. The TmAtg13 HORMA domain had a centrally-placed five-stranded antiparallel β-sheet centered on a α/β fold. The three-stranded β-sheets (β4′, β4″, and β8″) were placed on one end of the structure, whereas the α-helices packed the other side of the structure. The predicted TmAtg13 model showed homology with the spindle checkpoint protein Mad2, particularly the c-Mad2 conformation involved in binding other proteins at the spindle checkpoint (Figure 3Bi). All the TmAtg13 regions excluding the three-stranded β-sheet HORMA fold could be superimposed on the Mad2 structure (Figure 3Bii).

The TmAtg13 HORMA domain was comprised of 179 amino acid residues with a molecular weight of 20.1 kDa (pI, 8.91), and the molecular formula C_902_H_1456_N_238_O_261_S_11_. The putative phosphorylation sites for the TmAtg13 HORMA domain were serine (S23, S100), threonine (T31, T39, T71, T119, and T163), and tyrosine (Y102, Y133, and Y153) residues. TmAtg13 had a higher proportion of polar residues (45.2%) and basic amino acids (12.2%), which indicating that it favors a hydrophilic environment.

### TmATG13 phylogenetics study

**Figure 4:**
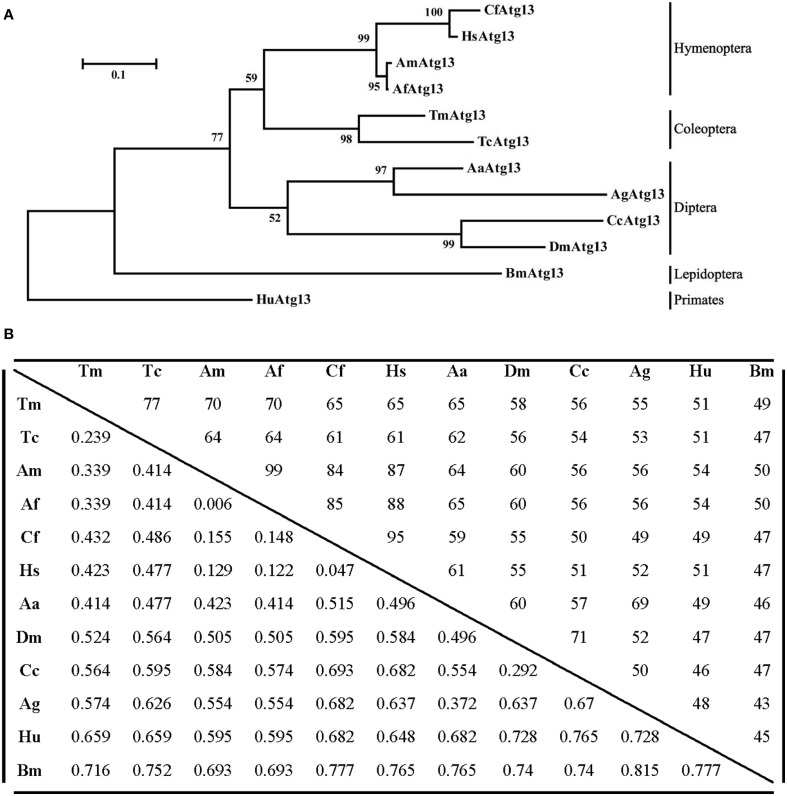
**Molecular phylogenetic analysis and percentage identity matrix for the TmAtg13 HORMA domain. (A)** An evolutionary tree for the TmAtg13 HORMA domain was constructed by using the maximum likelihood method and was based on the Jones- Thornton- Taylor matrix model. Bootstrap replications of 1000 were assigned and analyzed using the MEGA 6.06 program. The amino acid sequences with the GenBank accession numbers were as follows: TmAtg13; *Tenebrio molitor* Atg13, TcAtg13; *Tribolium castaneum* Atg13 (XP_968716.2), CfAtg13; *Camponotus floridanus* Atg13 (EFN61542.1), AmAtg13; *Apis mellifera* Atg13 (XP_623782.1), AfAtg13; *Apis florea* Atg13 (XP_003690771.1), HsAtg13; *Harpegnathos saltator* Atg13 (EFN82266.1), BmAtg13; *Bombyx mori* Atg13 (XP_004924339.1), AaAtg13; *Aedes aegypti* Atg13 (XP_001664333.1), AgAtg13; *Anopheles gambiae* Atg13 (XP_315727.4), CcAtg13; *Ceratitis capitata* Atg13 (XP_004523373.1), DmAtg13; *Drosophila melanogaster* Atg13 (NP_649796.1), and the outgroup HuAtg13; *Homo sapiens* Atg13 (AAH06191.1). **(B)** Percentage identity and distance matrix of TmAtg13 with its orthologs as analyzed using the ClustalX2 and MEGA 6.06 programs.

The TmAtg13 N-terminal HORMA domain was analyzed with the representative Atg13 sequences from its orthologs through a phylogenetics study using the neighbor-joining method. As shown in Figure 4A, TmAtg13 was clustered into the same branch as *Tribolium castaneum* Atg13 (TcAtg13) with a bootstrap percentage of 97%. The beetle Atg13 was clustered separately from the hymenopteran, dipteran, and lepidopteron cluster. Human Atg13 was considered as an out-group in the taxonomic classification of Atg13 sequences from insects. A percent identity and distance matrix was also designed to understand the level of sequence conservation among the *T. molitor* Atg13 sequences and its orthologs (Figure 4B). Identity of TmAtg13 was 77% with TcAtg13. In addition, the identity of the TmAtg13 protein sequence with hymenopteran orthologs from hymenoptera was in the order of 65–70%, followed by the dipteran ortholog at 55–65%.

### TmATG13 mRNA transcriptional profiles

**Figure 5:**
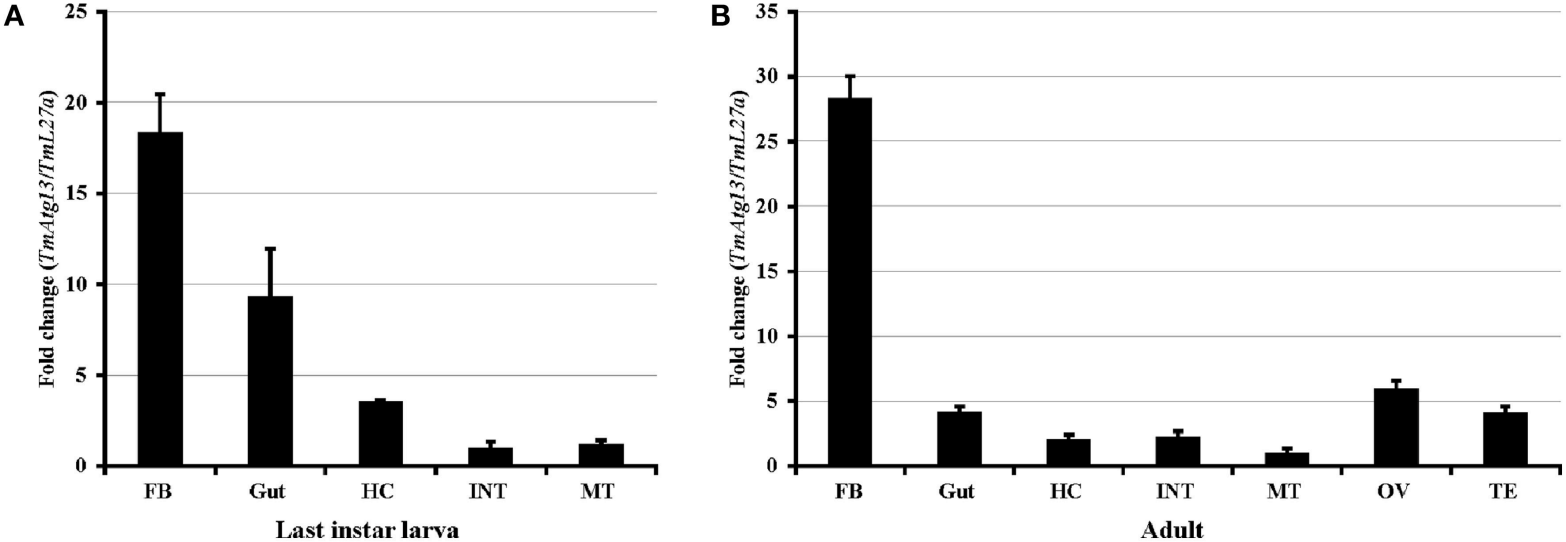
**TmAtg13 larval and adult-tissue specific expression profiles. (A) TmAtg13 in the last instar larval tissues. (B)** The distribution of the transcript in 2-day old adult tissues. Ribosomal protein L27a (*Tenebrio molitor*) was used as an endogenous control. Data are presented as mean ± standard error (*n* = 3). Abbreviations are as follows: FB, fat body; HC, hemocytes; INT, integument; MT, Malphigian tubules; OV, ovary; TE, Testis.

Real-time PCR was performed using TmATG13-specific primers to determine the TmATG13 gene expression profiles (Table 1). The quantities of TmATG13 PCR products were indexed to the levels obtained for the TmL27a gene, known to be constitutively expressed in *T. molitor*. Figure 5A shows the TmATG13 transcript expression in the last-instar larval tissues and Figure 5B shows the fold-changes in adult *T. molitor* tissues. TmATG13 expression levels in the fat body and gut of last-instar larvae were relatively higher than those in hemocytes, the integument, and the Malphigian tubules. Similar results were observed in the *T. molitor* adults, where the TmATG13 transcript expression was higher in the fat body than that in other tissues. High levels of TmATG13 mRNA transcripts were also noted in the ovaries and testis of healthy *T. molitor* adults.

**Figure 6:**
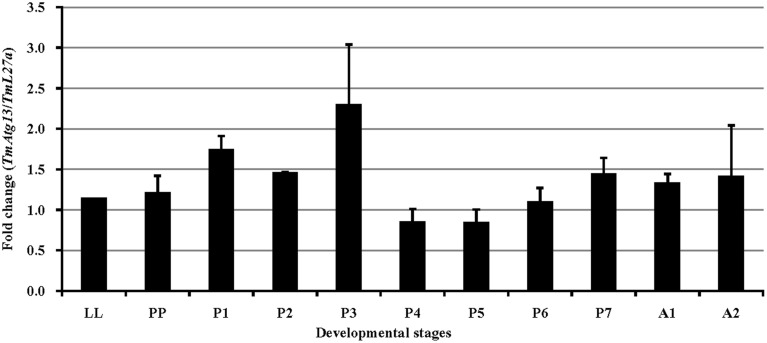
**Developmental expression patterns of TmAtg13 in the beetle, *T. molitor***. Ribosomal protein L27a (*T. molitor*) was used as an endogenous control. Data are presented as mean ± standard error (*n* = 3). Abbreviations are as follows: LL, last instar larva; PP, pre-pupa; P1–P7, 1–7 day old pupa, and A1–A2, 1–2 day old adult.

**Figure 7:**
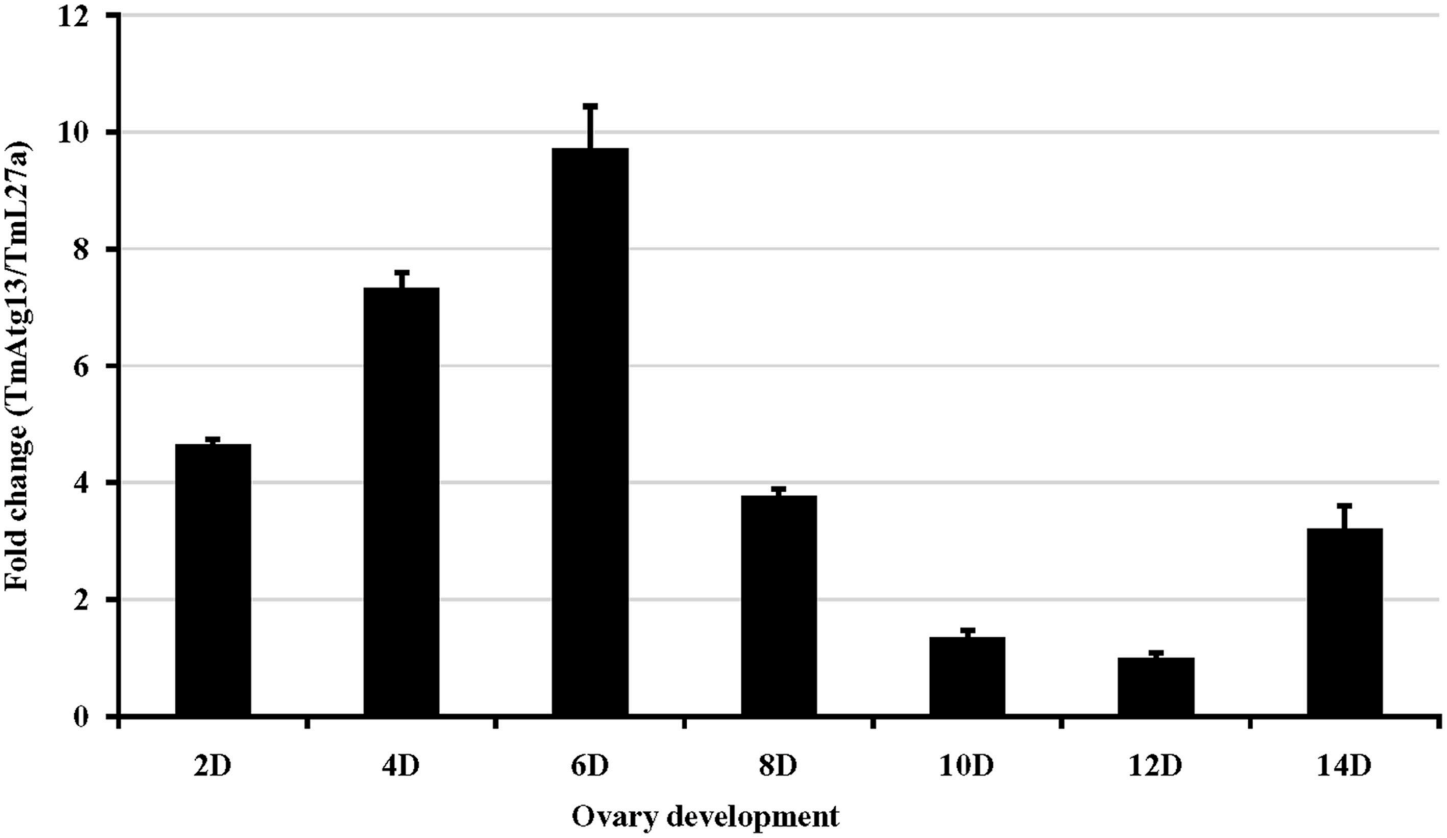
**Temporal expression of TmAtg13 during the maturation of adult ovary in the beetle, *T. molitor***. TmAtg13 mRNA expression levels are expressed as fold changes with respect to the endogenous control (ribosomal protein L27a). Expression of the mRNA transcripts was analyzed on alternate days (day 2, 4, 6, 8, 10, 12, and 14) of ovarian development. Data are presented as mean ± standard error (*n* = 3).

We also examined the TmATG13 expression at different developmental stages, including late larva, pre-pupa, pupal days 1–7, and adult day 1 and day 2 (Figure 6). The results suggest that TmATG13 mRNA is ubiquitously expressed, as no differences in the transcriptional profile were observed in between larval, pupal, and adult stages. We also detected the TmATG13 mRNA transcription profiles of TmATG13 mRNA during ovarian maturation in a time-course study (Figure 7). The TmATG13 mRNA expression levels of TmATG13 was greater early after adult eclosion, with the highest expression observed on at day 6 of development, followed by a significant decline during later stages of ovarian development and maturation.

### TmATG13 induction patterns and RNAi

**Figure 8:**
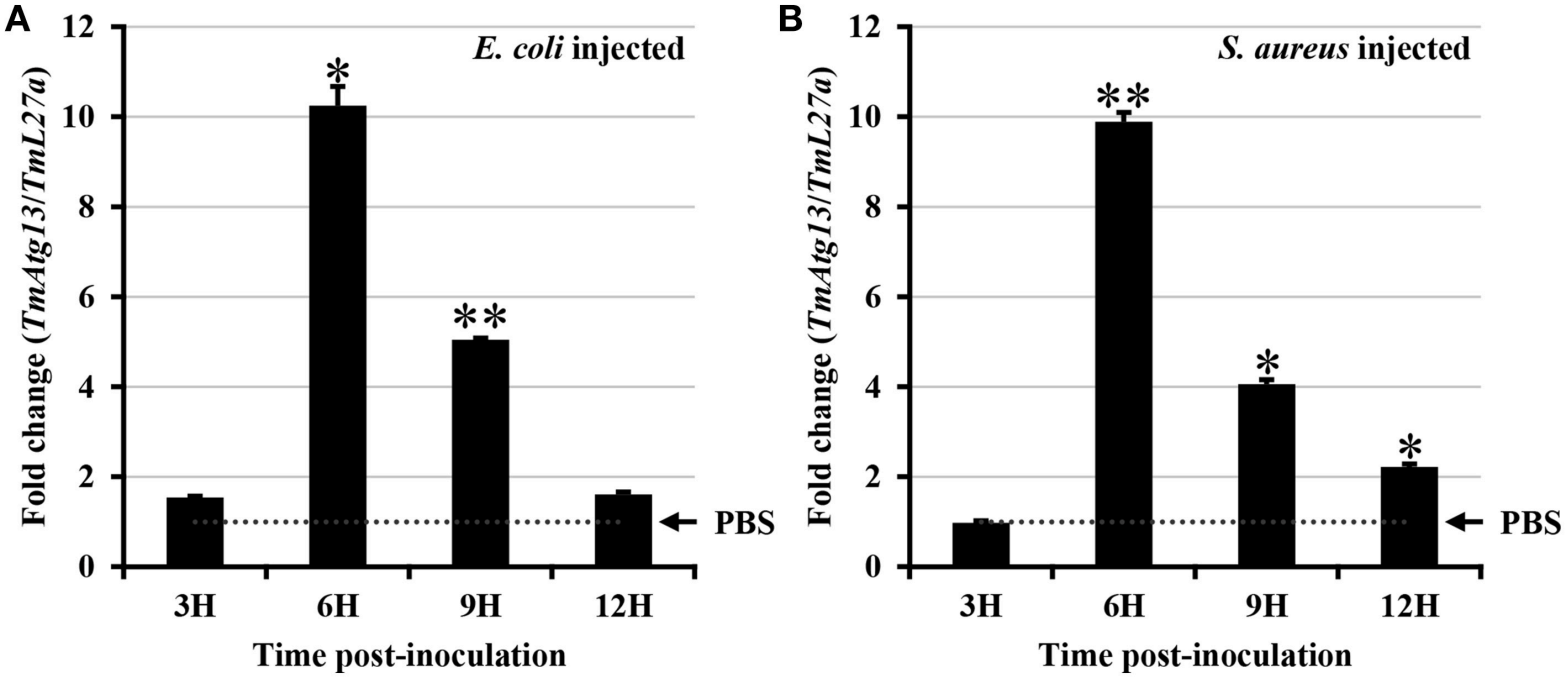
**Induction patterns of TmAtg13 in response to bacterial inoculation**. ***E. coli*** **(A)**, and ***S. aureus*** **(B)** were injected into *T. molitor* larvae and total RNAs were isolated at 3, 6, 9, and 12 h post-injection of microorganisms. Primers for *ribosomal protein L27a* from *T. molitor* (TmL27a) were used as endogenous control. Results of triplicate experiments have been provided with standard errors. ^*^ *P* < 0.05; ^**^ *P* < 0.01 (SAS, ANOVA).

**Figure 9:**
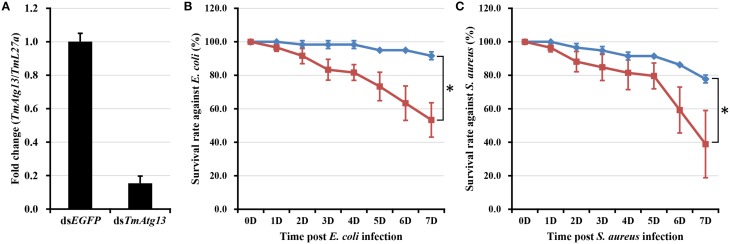
**RNAi-based silencing of TmAtg13 transcripts and survival assay. (A)** RNAi efficiency of TmAtg13 mRNA in dsTmAtg13 injected larvae compared with ds*EGFP*-treated *T. molitor*. Survival rate against *E. coli* **(B)** and *S. aureus* **(C)** measured in TmAtg13 silenced *T. molitor* larvae compared with dsEGFP-treated *T. molitor* larvae. Data are presented as mean ± SE. ^*^, significant (*P* < 0.05; Wilcoxon-Mann-Whitney test) change in the larval survivability was observed in between the dsEGFP and dsTmAtg13 silenced larvae.

*E. coli* and *S. aureus* were injected into *T. molitor* larvae to understand the TmATG13 induction pattern in response to microbial challenge, and TmATG13 transcript levels were monitored in a time-dependent manner. The results indicate that TmATG13 transcript levels increased steadily until 6 h compared to those in PBS-treated larvae, followed by a persistent decline at 9 and 12 h post-injection of *E. coli* (Figure 8A) and *S. aureus* (Figure 8B). The role of TmATG13 in survival of *T. molitor* larvae was investigated by knocking down the transcript levels followed by injections of *S. aureus* and *E. coli*. The RNAi efficiency measured after dsTmATG13 injection was found to be about 82% (Figure 9A). The results indicated that at 7 days post-injection, the survivability against *E. coli* and *S. aureus* was decreased by 39 and 38%, respectively, in dsTmAtg13-injected larvae compared to that in the dsEGFP-injected control groups (Figures 9B,C).

## Discussion

We screened a full-length ATG13 ORF from a *T. molitor* transcriptome database. Seven putative ATG genes, including ATG13, were identified previously from the tick, *Ixodes scapularis*, and all are involved in formation of autophagosomes (Van Zee et al., 2007). As expected, the TmAtg13 homolog characterized in the present study, showed high identity with Atg13 from *T. castaneum*, when compared with other insect Atg13 orthologs. We used the conserved amino acid sequence of the TmAtg13 N-terminal domain to model the protein structure in detail. It was found that the N-terminal Atg13 domain was unstructured and less conserved. This result agrees with other reports on the structure and function of Atg13 in model organisms. This unstructured Atg13 region is necessary and sufficient for binding to both Atg1 and Atg17 (Kabeya et al., 2005; Ragusa et al., 2012). This region is also a locus for the presence of regulatory Atg13 phosphorylation sites and conforms to our TmAtg13 observation. A potential human Atg13 ortholog shows about 15% identity with the *S. cerevisiae* Atg13 protein over their C-terminal amino acid sequence and 43% identity with the *Drosophila* Atg13 protein (Chang and Neufeld, 2009). Additionally, the Q-rich region, which is a characteristic feature of yeast Atg13, is not conserved in other species including *Drosophila, Tenebrio*, and human Atg13. Most of the studies have addressed the requirement of the unstructured Atg13 region to bind Atg1 through hypophosphorylation; a process essential to the translocation of this complex to the PAS.

Comparatively little is known about the structural features of conserved Atg13 domain, even though the 2.3 Ǡ crystal structure of LtAtg13 has been solved with structural similarity to the spindle checkpoint protein Mad2 (Mapeli et al., 2007; Chao et al., 2012). Mad2 has two different stable conformations, and O-Mad2 and C-Mad2, with C-Mad2 conforms completely to the HORMA domain (Aravind and Koonin, 1998). The LtAtg13 HORMA fold domain is required for autophagy and to recruit the Atg14 of phosphatidylinositol (PtdIns) 3-kinase subunit Atg14 but not to localize Atg13 (Jao et al., 2013a,b). The amino acid sequences corresponding to the N-terminal *T. molitor* Atg13 domain were used to construct a homology model based on the crystal structure of *L. thermotolerans*. The predicted TmAtg13 model of TmAtg13 showed all the secondary structural features, including the Mad2-related organization, and the safety belt region. The HORMA fold structure toward the end of the N-terminal region which is unique to Atg13 protein of *T. molitor*. In this regard the putative existence of TmAtg13 HORMA domain as a phosphorylated conformational switch needs to be addressed further. The concept of phosphoregulated switch in Atg13 in *L*. *thermotolerans* needs to be further investigated. It has been shown that a mutation in the putative phosphate sensor in the HORMA domain does not show a distinct autophagy phenotype (Jao et al., 2013a) although mutations to TOR phosphorylation sites in Atg13 to Ala lead to constitutive autophagy (Kamada et al., 2010).

Real-time PCR- based quantification was useful to measure the expression of TmATG13 transcript in healthy *T. molitor* larval and adult tissues as well as during various developmental stages. We observed enhanced mRNA expression of TmATG13 in the fat body of *T. molitor* larvae and adults. In general, autophagy is rapidly induced by starvation in the larval fat body as shown in previous reports (Scott et al., 2004; Umemiya et al., 2007; Malagoli et al., 2010). In fat body cells of *Drosophila*, overexpression of Atg17 leads to phosphorylation of Atg13 that enhances autophagy in an Atg-1 dependent manner (Nagy et al., 2014). Midgut cells have also been examined for autophagosomes or autophagic vacuoles under starved conditions (Tarnowski and Coons, 1989). Lysosome number and the quantity amount of lysosomal enzymes increase during starvation for autophagy-related processes (Vilaplana et al., 2007).

The high mRNA expression of TmATG13 in the *T. molitor* larval gut was a positive signal for specific degradation of midgut epithelial cell components. We are working toward generating TmAtg13 antiserum to determine the location of this protein in fat body and midgut cells of unfed *T. molitor* larvae by immunohistochemistry and immunoblotting. Hyperphosphorylated and dephosphorylated Atg13 species have been detected recently by Western blot (Miller-Fleming et al., 2014). The consistent and ubiquitous Atg13 transcriptional profile during *T. molitor* development may suggest a routine involvement of autophagy in the degeneration of larval tissues and organs, and a temporal transition in pupal and adult metamorphorhic stages. Autophagy during lepidopteran metamorphic transition periods is well characterized, particularly in the moths *Heliothis virescens* (Tettamanti et al., 2007a), *Alabama argillacea* (de Sousa et al., 2009), and the butterfly *Pieris brassicae* (Tettamanti et al., 2007b). A lower TmATG13 expression level during the late stages of normal ovarian development suggests impaired autophagy, although this needs to be confirmed by TOR activity and autophagosomes/autolysosomes production. Also there is a possibility of the activation of a programmed cell death (PCD) process where the features of autophagy and apoptosis are concomitant. A report in this direction states that autophagy largely controls cell death in *D. melanogaster* oocytes by regulating the development of nurse cells and DNA fragmentation in late oogenesis. This is because a genetic inhibition of autophagy by removal of function of ATG1, ATG13, and VPS34 results in persistent nurse cell nuclei without fragmented DNA (Nezis et al., 2010).

The increase in TmATG13 transcripts shortly after hours of *E. coli* and *S. aureus* infection in *T. molitor* larvae indicates pathogen clearance through the autophagic process. This observation is in logic with the reduced survivability of TmATG13-silenced larvae in *E. coli* and *S. aureus* infected *T. molitor* whole larva. Previous reports have documented the direct role of autophagy in restricting replication of *Listeria monocytogenes, Shigella flexneri, Salmonella typhimurium*, and *Mycobacterium tuberculosis* (Gutierrez et al., 2004; Birmingham et al., 2006; Yano et al., 2008; Mostowy et al., 2011) in infected cells. Atg13 protein is required for phagosome formation in the hierarchical, canonical autophagy, and therefore a depletion of TmAtg13 via RNAi could have reduced the ability of the larvae to contain the pathogens through autophagy.

In summary, we identified an ATG13 homolog from the *T. molitor* transcriptome database and characterized its sequence and structural features using a bioinformatics approach. The conserved N-terminal Atg13 domain conformed to the HORMA domain architecture of human Mad2 protein and Atg13 protein of *L. thermotolerans*. We found the expression of ATG13 at the level of RNA in different developmental stages and tissues of *T. molitor* larvae and adult. However, we did not confirm its existence morphologically. In preparing RNAi screen for TmAtg13 and delineating its putative role in autophagy-mediated clearance of bacterial pathogens such as *E. coli* and *S. aureus*, we are close to providing a nexus for study of the individual structural components of the HORMA domain in regulation of autophagy. Furthermore, the dynamics of TmATG13 interaction with Atg1 and Atg14, especially in midgut tissues is under study to understand the insect's starvation strategy.

## Author contributions

Conception and design of work: YL, YH. Obtainment of experimental data: JL, YJ, KP, BP, HT, GS, RC. Writing and revision of manuscript: BP, RC, YH.

### Conflict of interest statement

The authors declare that the research was conducted in the absence of any commercial or financial relationships that could be construed as a potential conflict of interest.
